# Improving the protein quality of New Zealand vegan diets: an optimisation modelling approach incorporating energy constraints and diet acceptability

**DOI:** 10.3389/fnut.2026.1807755

**Published:** 2026-04-22

**Authors:** Bi Xue Patricia Soh, Matthieu Vignes, Nick W. Smith, Pamela R. von Hurst, Warren C. McNabb

**Affiliations:** 1 Sustainable Nutrition Initiative, Riddet Institute, Massey University, Palmerston North, New Zealand; 2 School of Mathematical and Computational Sciences, Massey University, Palmerston North, New Zealand; 3 The New Zealand Institute for Bioeconomy Science Ltd (Plant and Food Group), Palmerston North, New Zealand; 4 School of Sport Exercise and Nutrition, College of Health, Massey University, Auckland, New Zealand

**Keywords:** amino acids, dietary optimisation, linear programming, protein quality, vegan diets

## Abstract

**Introduction:**

Under consumption of certain indispensable amino acids (IAAs) is common in poorly planned vegan diets, but targeted dietary modifications through optimisation modelling can improve the overall protein adequacy and protein quality of these diets.

**Methods:**

Shortfalls in protein and IAAs in the existing diets of a sample of New Zealand vegans (stratified into three clusters with varying protein intake) were calculated by comparing daily intakes to individual requirements. An energy-tailored optimisation using linear programming was used to add protein-rich foods present in each individual’s current diet to meet all protein and IAA requirements while respecting serving-size constraints and remaining within individual energy boundaries. When daily diets exceeded upper energy constraints, energy-dense and low-protein foods were identified and removed to accommodate the addition of protein-dense foods. Post-optimisation analysis assessed changes in intake of protein, IAAs, dietary fibre and selected micronutrients, with results compared across clusters.

**Results:**

Protein and IAA shortfalls were more prevalent in cluster 1 (85% of daily diets) compared to clusters 2 (61.1%) and 3 (30.8%). Legumes and pulses contributed most to total protein and lysine at lower energy costs, while nuts and seeds contributed most to methionine and leucine, but at higher energy costs. Optimisation resolved shortfalls using habitual diets in 90% of the daily diets. Post-optimisation micronutrient analysis showed continued risks of shortfalls for calcium, vitamin B12 and iodine.

**Discussion:**

Mathematical optimisation can enhance the protein adequacy and protein quality of vegan diets while preserving some individual acceptability. However, full adequacy remains challenging in energy-constrained diets with large deficits in protein and IAAs.

## 1 Introduction

Foods are not equal in their nutrient provision. For example, the quantities of utilisable indispensable amino acids (IAAs) are generally lower in plant-sourced proteins as compared to animal-sourced proteins (1). This disparity is particularly consequential in vegan diets, where the exclusion of all animal-sourced foods means that all protein must come from plant-based (PB) sources. PB protein sources can be strategically combined to complement one another’s amino acid (AA) profiles, hence mitigating shortages of these IAAs in individual foods (2). Food choices are however governed by various factors, including cultural acceptability, individual preferences, and cost of foods, leading to the emergence of varied dietary patterns that differ in diet quality and nutritional profiles.

Devising vegan diets that are both acceptable and high in protein quality can be challenging in situations when food group (FG) diversity is limited or dominated by energy-dense and nutrient-poor foods. Such combinations restrict the extent to which a diet can be modified to meet requirements without substantial food substitutions (3). Improvements in diet quality must consider acceptability and adherence within the population. Defining acceptability constraints is however challenging due to the subjectivity associated with individual food preferences and cultural practices (4).

Mathematical optimisation using linear programming (LP) offers an efficient approach to identify the most suitable combinations of available foods that can satisfy nutritional constraints while minimising deviation from the original diet (3, 5, 6). Past modelling techniques have incorporated various strategies to ensure dietary realism, such as setting upper and lower bounds for food intake (3, 4), imposing penalties for deviations from current energy and serving-size intakes (4, 7), using each individual’s current dietary intake as the baseline (3, 8) and benchmarking against healthier diets within the same population group (4, 9). LP can thus be applied in the design of high protein quality vegan diets by measuring the extent to which total protein and utilisable IAA intake can be obtained within the boundaries of individual energy requirements while implicitly accounting for dietary preferences by incorporating some of the above acceptability constraints.

Previously, lysine and leucine deficiencies were identified among a sample of New Zealand vegans, where approximately half of the cohort was below the daily requirements for these IAAs (10). Analysis at the level of the eating occasion (EO), where foods consumed at the same time point were categorised into the same EO, revealed three distinct clusters which varied in the quantity of protein and IAA intake Only a small percentage of daily diets (15.7%) from one cluster was close to achieving the total protein requirement at the level of the EO (11). Taken together, a large proportion of the current vegan diets in the sampled NZ cohort were at risks of protein and IAA deficiency and should be modified through LP to bridge these gaps. The extent of modification necessary is hypothesised to vary across individuals and clusters, with daily diets nearer to sufficiency, requiring minimal changes, in comparison to those with significant deficits in protein or IAAs.

Prolonged deficiencies in AAs can impair metabolic functions in the body, including protein synthesis and muscle mass maintenance, with certain populations, such as the elderly, being at higher risks (12). It is often more challenging to deliver comparable protein quality within the same energy allowance as many PB foods have lower protein-to-energy ratios than animal-sourced foods (13, 14). The challenge may be compounded by higher fibre intake from PB foods, which promote satiety (15), and the concurrent need to maintain micronutrient adequacy if supplementation is absent. While past studies have examined substitutions of animal-sourced proteins with PB proteins in varying proportions (16–18), no studies have applied optimisation methods to systematically improve deficient vegan diets to overcome shortfalls in protein and IAA. Given the critical roles of IAAs in metabolic health and the well-recognised challenges of achieving adequate protein quality within energy boundaries, this study provides a timely and practical contribution.

The overall aim of this study was to generate dietary modifications based on each individual’s existing daily food intake, with the goal of rectifying shortfalls in total protein and IAA, commonly observed in vegan diets, within individual energy limits. By incorporating familiar foods into the diet, this individualised framework allows diet modifications to remain culturally and behaviourally relevant to each individual (3). A secondary aim is to quantify the deviation in food intake and nutrient contribution required to achieve adequacy by comparing the modified and baseline diets of the population and how these vary across the different clusters. Modified diets should be feasible in implementation, so the application of Food-Based Dietary Guidelines (FBDGs), such as for serving sizes (6) and energy constraints, serve as boundaries that promote the increased likelihood of real-world compliance.

While micronutrient adequacy is highly relevant in vegan diets, it was not included as primary constraints. Micronutrient adequacy in vegan diets can be achieved through supplements or fortified foods, but neither of these was comprehensively examined in this study. Hence, without adequate supplementation data, requiring that all nutrients be met may produce a narrow margin for successful optimisation. Instead, a post-hoc evaluation of micronutrients was conducted to determine if the protein-focused optimisation produced micronutrient co-benefits.

## 2 Methods

### 2.1 Study design and data collection

Dietary data were collected as four-day food diaries from 193 vegans through the Vegan Health Research Programme, a cross-sectional study which examined relationships between vegan dietary patterns and nutrition and/or health outcomes. Data was collected at the Human Nutrition Research Unit at Massey University, Auckland. Ethical approval was granted by the Health and Disability Ethics Committee (HDEC 2022 EXP 12312), and written informed consent was obtained from all participants prior to data collection. As part of the inclusion criteria, only healthy adults aged 18 and above who were not pregnant or breastfeeding and had been following a self-reported vegan diet for at least 2 years were recruited.

Food items listed in the food diaries were processed with the FoodWorks Professional nutrient analysis software (Xyris, Australia 2022) and matched to appropriate foods found in FOODfiles, the NZ food composition database (19). This provides the nutrient composition, such as total protein, dietary fibre, vitamins, and minerals, for each food item. AA composition is not currently available in FOODfiles. Hence, to quantify the IAA content in NZ foods, each food item was matched to a similar food and cooking method from the food and nutrient composition database of the United States Department of Agriculture (USDA) (20) and normalised to the protein content of NZ foods. In cases where an exact match was unattainable, the food item was matched to the closest available items to calculate average values for protein and IAA. To improve the quality of matches, the food matching guidelines from FAO/INFOODS were followed (21). To indicate the bioavailable proportion of protein and IAA, food composition values were adjusted using available True Ileal Digestibility (TID) coefficients, where TID data is available (22–25). Food items were assigned to broader or closely matched FGs with available TID values so that the optimisation inventory contained foods where the protein and IAA contents were already corrected for digestibility. IAAs with currently available TID values for a large range of plant foods (histidine, leucine, lysine, cystine, methionine, sulphur-amino acids (SAAs), threonine and tryptophan) were included in this study. Selected foods would therefore more accurately reflect the utilisable protein and IAA available to meet requirements.

As described in the introduction, the dietary data was subdivided into three distinct dietary patterns varying in protein intake and protein quality at the level of the EO (11). After excluding days with missing consumption or time data, a total of 766 daily diets were included to quantify daily nutrient shortfalls relative to individual requirements. These represented the baseline diets for modifications.

### 2.2 Calculation of individual nutrient requirements and shortfalls

Individual daily protein requirements were calculated based on sex, age and body weight (BW), provided by the Nutrient Reference Values (NRV) for Australia and New Zealand (26). The Estimated Average Requirement (EAR) values were used for this computation. This was 0.68 g/kg of BW for males and 0.60 g/kg for females of between 19 to 70 years of age. Individual daily IAA requirements were calculated based on individual BW, using the reference values for adults aged 18 years and above, from the Food and Agriculture Organisation (FAO) (25) (See Supplementary Table S1).

The minimum daily energy requirement in megajoules (MJ) for each individual was calculated by accounting for the sex, age, BW and height, as well as the physical activity level (PAL) for each individual (26). (See Supplementary material for calculation of the PAL). The Estimated Energy Requirement (EER) value was then assigned to each individual based on sex, BW, height, age, and the calculated PAL (26). While an upper limit for dietary energy intake has not been established (26), a 20% increase above the EER was applied as a pragmatic upper boundary. This threshold was informed by preliminary testing (see Sensitivity Analyses) of smaller incremental increases and considerations of feasible dietary modifications. This allowance provided the model with greater flexibility to choose a wider range of foods for individuals who met or exceeded their EER but had significant shortfalls for protein and/or IAAs. Shortfalls in protein, IAA, and energy [for individuals with daily intake < minimum requirement for EER (EER_min_)] were tabulated for each day by subtracting each nutrient’s total daily intake from the daily requirement intake of each nutrient.

**Figure 1:**
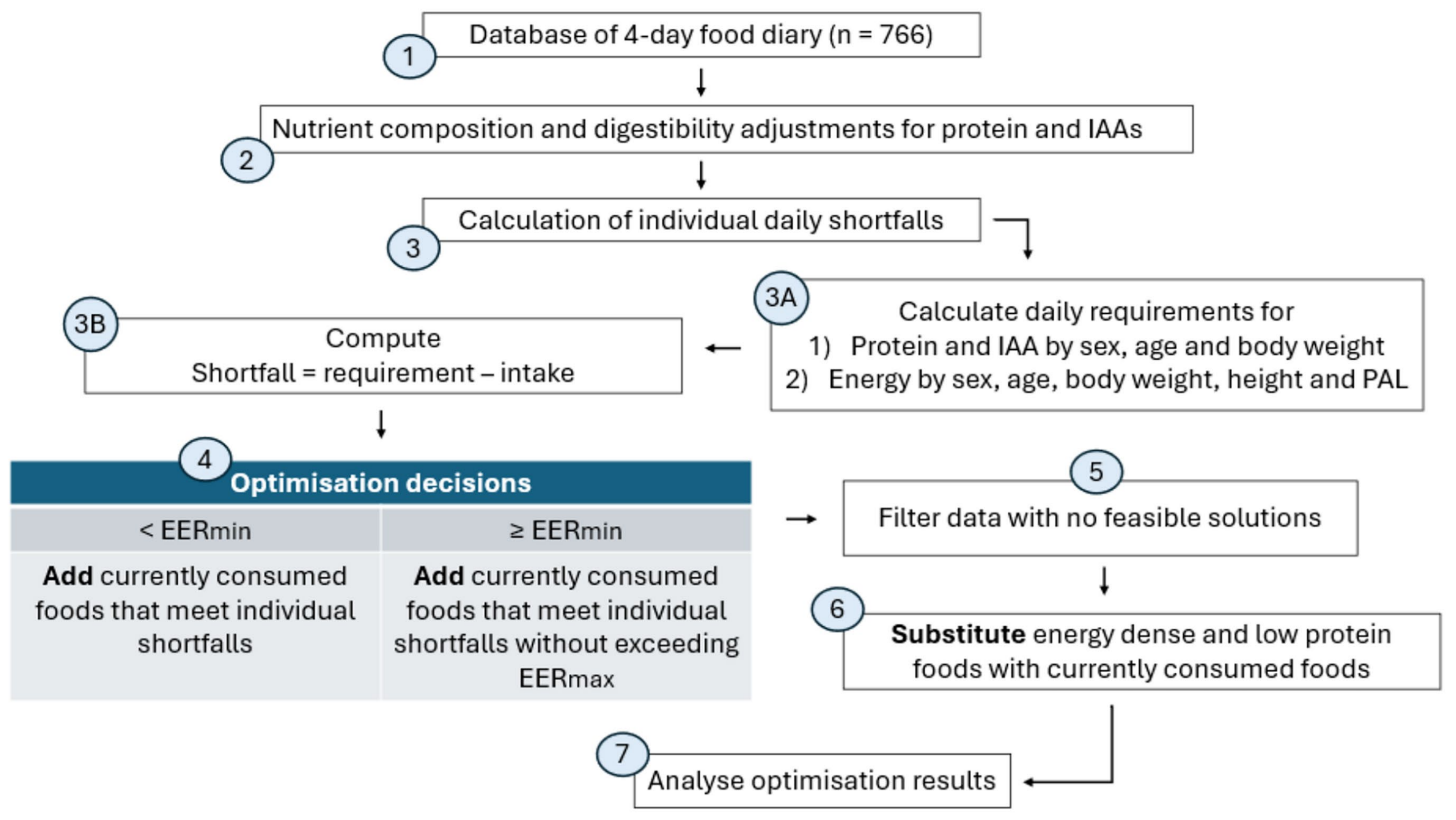
Methodology of LP optimisation IAA, indispensable amino acids; PAL, physical activity level, EER_min_, minimum estimated energy requirement per day, EER_max_, maximum estimated energy requirement per day. Numbers indicate the steps taken in the optimisation process. (1) Dietary data was in the form of 766 self-reported food diaries, from a cohort of 193 NZ vegans. (2) Each food item was matched to its nutrient composition. Total protein and IAAs were adjusted for digestibility. (3) Shortfalls were calculated for each of the 766 daily diets by first, (A) calculating the daily requirements for energy, protein and IAA, with demographic and anthropometric data of each vegan and (B), computing the daily shortfalls. (4) LP optimisation methods depended on the baseline energy intake of the daily diets. Should energy intake be below (<) EER_min_, energy formed part of the shortfall to be filled, whereas when intake met or exceeded (≥) EER_min_, foods could only be added within the residual energy allowance up to the EER_max_ (5) Combination of cases that exceeded the EER_max_ with other unsuccessful cases (6) For these cases, a substitution strategy was used, to provide energy headroom, for further food additions (7) All cases with feasible solutions from step 4 and step 6 were analysed for nutrient intake after optimisation.

Figure 1 presents the overall methodology, highlighting the processes in the database preparation and subsequent diet optimisation.

### 2.3 Diet optimisation using LP

One of the constraints of the LP model was that foods added should fulfil any protein and IAA shortfalls. Hence, for each nutrient *i* (where *i* may be TID-adjusted protein or one of the TID-adjusted IAAs), the quantity *x_j_* (in grams) of all foods *j* in the optimised diet must together supply at least the daily requirement, *R*_i_. Formally:


$$\;\;\sum_{j\in J}a_{\mathrm{ij}}x_{j\;\;}\geq R_{i}$$ (1)

for each nutrient *i*, where:

*a_ij_* is the quantity of nutrient *i* per gram of food *j*,

*R_i_* is the daily nutrient requirement for nutrient *i*.

*J* represents the set of all possible food items in the optimised diets.

Foods in the optimised diet must result in an energy contribution that is within the lower (EER_min_) and upper (EER_max_) energy boundary for that individual. This is expressed as:


$$\;\;\;\mathrm{EE}R_{\min}\leq \sum_{j\in J}e_{j}x_{j}\leq \;\;\mathrm{EE}R_{\max}$$ (2)

*e_j_* represents the energy (MJ) per gram of food selected, *x_j_.*

As shown in Figure 1, the optimisation decision depends on the daily energy intake (step 4). In all cases, the model is constrained to not exceed EER_max_. If the daily diet does not meet the EER_min_, then energy is falling short of what is required to support metabolic function, and the model chooses foods that will simultaneously cover the shortfalls for protein, IAA and energy.

The model may not return feasible solutions in every case (step 5). For example, if the current energy intake is close to EER_max_ but significant deficits exist for protein and IAA, it may not be possible to add foods that fill the protein deficit while respecting the energy constraint. A manual substitution strategy was then utilised to identify and remove the most energy-dense, but protein-poor foods from the diet, to accommodate the addition of foods that can fill nutrient gaps (step 6).

To do so, an energy-to-protein ratio was calculated for each food in these cases. The first substitution strategy involved removing 25% (by mass) of the original foods that had the highest energy-to-protein ratio, recalculating the new shortfalls and re-conducting the LP model to add foods. Cases which still had no feasible solutions were then identified. The top 50% of the original foods that had the highest energy-to-protein ratio were then removed, and the food addition process was performed again. This approach allowed direct identification of foods that were suboptimal contributors to protein quality while remaining within the energy constraints of a vegan diet. Any remaining cases without feasible solutions were not addressed further, since removing >50% of food from the baseline diet was considered an unacceptably large departure from habitual intake. Beyond this point, additional removal may not yield meaningful gains in energy alignment, nutrient contributions and food diversity, and risked producing unrealistic solutions reliant on high amounts of limited number of foods (e.g., high servings of nuts and seeds). Substantial dietary restructuring, ideally supported by practical dietary guidance may then be necessary to improve protein quality while maintaining acceptability.

An acceptability constraint was implicitly introduced through the proxy of adding foods already in an individual’s diet over foods from outside their existing diet. Food items already part of each individual’s diet were available for selection by the LP model, except for certain foods, which were intentionally omitted at this point. For example, Brazil nuts were removed due to their high potential in exceeding selenium upper limits (one seed from a high-selenium area is sufficient to meet the daily selenium allowance) and the narrow window between selenium deficiency, adequacy and toxicity (27). Seaweed, which has a wide range of iodine content depending on species (0.02 to 428 μg per gram across species in this study’s inventory), could exceed the upper limit of iodine intake (28) and were also excluded. Herbs and spices are mostly used as condiments and were not considered as feasible foods to address protein shortfalls in the diet.

The objective function of the LP model was to minimise the total weight of food added to the diet, thereby limiting the addition of large volumes of food that cannot be administered in realistic diets. Weight-based constraints corresponding to serving sizes were set for each FG and introduced as continuous variables to prevent excessive addition of each food item, as guided by the NZ Eating and Activity Guidelines (29) and listed in Table 1.

**Table 1:** Weight of food per serving size for each food group.

Food group	Maximum weight (g) per serving
Fruit	150
Grains and pasta	120
Legumes and pulses	150
Nuts and seeds	30
Potatoes, kumara and taro	75
Sugar and sweets	30
Vegetables	75
Yeast and condiments	30

The serving constraint for spirulina was capped at 3 g, according to recommended guidelines from the literature (30, 31). To encourage dietary diversity, the LP optimisation was constrained to adding up to one serving for each unique food item (but not FG). For example, a maximum of 30 g of sesame seeds and 30 g of cashew nuts could be added to address shortfalls. The characteristics of the optimisation model are described in Table 2.

**Table 2:** Characteristics of optimisation model.

Constraints	Objective function	Decision variable	Solution
1. Meet protein and IAA requirements	Minimise weight of food added	Food items reported in any of the 4 days in each individual’s food diary	Weight, energy and nutrient contribution of food items added
2. Serving size per food item, with maximum of one serving per food item			
3. Optimised diet does not exceed EER_max_			

Optimisation was conducted separately for all individual diets in each cluster within the vegan cohort, and the solutions were compared across clusters. The LP optimisation was completed using R Studio (version 4.3.1) with the *lp solver package* (32).

### 2.4 Data analysis: post-optimisation assessment of total protein and IAA

Results were analysed (Figure 1, step 7) and compared across clusters 1 to 3. The mean weight in grams of added foods for each FG was tabulated. The mean contributions to energy, protein and IAA from each added FG were computed as a percentage of the minimum daily requirement. To obtain the final nutrient content after optimisation, the quantity of nutrients from foods introduced by optimisation was added to the baseline nutrient content. For cases where foods had to be substituted (Figure 1, step 6), the diets after removing energy-dense foods were used as the new baseline diets for this computation.

The baseline diets (dietary data in step 1 of Figure 1) and the final optimised diets for all individuals were then compared to determine how the contributions of energy, protein and IAA per FG changed after the diet modification. The total nutrient content for the baseline and final diets was also computed as a percentage of the minimum daily requirement. The final protein and IAA content for all optimised diets were computed per individual BW (kg) and compared with reference values. For total protein, the final quantity was compared to the EAR and the Acceptable Macronutrient Distribution Range (AMDR), which is between 10% to 35% of caloric intake (26, 33).

### 2.5 Data analysis: post-optimisation assessment of changes in other nutrient levels

Finally, dietary fibre, alpha-linolenic acid (ALA) (the most prominent long-chain fatty acid in plant-sourced foods) and selected micronutrient quantities of the optimised diets were computed to assess how these nutrients changed from baseline. Vitamin B12, calcium, iodine, iron and zinc were the micronutrients focused on in this study because of increased risks of deficiency associated with a vegan diet (34–36). Total sodium content in the optimised diets was also analysed against reference values to check excess sodium. The EAR or adequate intake (AI), when EAR is not available, was used as the minimum daily requirement (26). The UL per day was also provided when applicable (26). (Supplementary Table S2). Note that these values were not used as constraints in the optimisation; they are included as further contextual information about the quality of the optimised diets. Post-optimisation evaluations were conducted to assess the potential for modified diets to exceed the safe upper limits of micronutrients.

## 3 Results

### 3.1 Optimisation results with targeted food additions and substitutions

The number of daily diets with at least one shortfall in cluster 1 was 250 (total of 294 daily diets), cluster 2 was 215 (total of 352) and cluster 3 was 37 (total of 120). More severe shortfalls, with simultaneous shortfalls in protein and all IAAs, were observed in 10% of daily diets in cluster 1% and 4% in cluster 2. No daily diets in cluster 3 showed concurrent shortfalls in protein and all IAAs. Lysine, leucine, and methionine had the largest average deficits compared to their daily requirement. The first round of LP optimisation yielded feasible solutions for 303 out of 502 daily diets. For the remaining 199 daily diets, 100 of these had already exceeded the EER_max._at baseline and could not accommodate further additions. The remaining 99 also had insufficient residual energy capacity even though they were below the EER_max_ (mean of 1.07 MJ).

Hence, food substitution was conducted for these 199 cases. When the top 25% of foods by energy-to-protein ratios were removed before optimisation, feasible solutions were obtained for 118 of the 199 cases. Foods removed were predictably higher in energy: fruits, savoury sauces, grains, and sugary foods and beverages. Before optimisation, these diets had a mean energy of 3.09 MJ below the EER_max,_ and relatively smaller shortfalls. Contrastingly, for the remaining 81 unsolved cases, the diets had a mean energy of 2.14 MJ below the EER_max_ but with larger shortfalls, especially for protein. For these 81 cases, further food substitutions were required. The top 50% of foods with the highest energy-to-protein ratios were removed to provide even higher residual energy capacity (mean 4.20 MJ) for new food additions. After this substitution, the LP model was able to find feasible solutions for 29 of these cases but 52 cases had no feasible solutions.

Overall, the optimisation process obtained solutions for 90% of daily vegan diets which had at least one shortfall at baseline. This was 219 cases in cluster 1, 195 in cluster 2 and 36 in cluster 3. Of the cases where no feasible solutions could be obtained from the current diet (*n* = 52), cluster 1 diets formed the largest proportion at 60%, followed by cluster 2 (38%). These diets may benefit from other modification strategies (explored in the sensitivity analyses).

The mean mass of total food added per person per day was highest for clusters 1 and 2, at 44.9 g (SD = 26.4) and 43.7 g (SD = 27.3), respectively, while the mean mass added for cluster 3 was 39.3 g (SD = 25.8). The mean number of unique foods added for each individual was again higher in clusters 1 and 2, at 3.47 and 3.24 items, respectively, while for cluster 3, 2.80 items were added. The most frequently added food items were from the FGs, “legumes and pulses” and “nuts and seeds,” with peanut, pumpkin seed, almond, plant-based meat alternatives (PBMAs), tofu and pea protein isolate (such as in protein powdered beverages) the most commonly added foods in all clusters.

In all the clusters, legumes and pulses contributed most to total protein and IAAs such as threonine, lysine and histidine while providing the smallest proportion of energy. While nuts and seeds contributed more energy per gram of added food, their contribution to total protein, tryptophan, methionine, leucine and SAA was higher than that of legumes and pulses. Yeast-containing food items were noted to contribute to lysine with small increments in energy. The contribution of novel plant-based alternatives (PBAs) to energy and protein was approximately 10–15% for both protein and IAAs, and <10% for energy across all clusters. As fruits were added only in a few cases (two in cluster 1 and four in cluster 2), the average quantity added was inflated, driven by large portions (up to 150 g per fruit) in a few individuals. Fruit, as well as sugar and sweets, was added only to individuals who had an energy shortfall. Given their relatively high energy content, these foods may have been added to close the energy gap when the serving-size limits for other FGs—nuts and seeds, legumes and pulses—have reached the maximum serving limit.

Table 3 presents the mean contributions of food weight, protein, and IAA as percentages of daily requirements for all added food items (categorised into respective FGs) for cases in which LP optimisation could find solutions. Large standard deviations (SD) indicated large variation in the quantities of food items added across daily diets.

**Table 3:** Mean added weight in grams (SD) and mean percentage contribution to energy, protein and IAA from each food group (FG) across clusters.

Food group	Mean weight in grams (SD)	Energy and nutrient contribution to minimum daily requirement^1^ (%)									
		Protein	Tryp	Thr	Leu	Lys	Met	Cys	SAA	His	Energy
Cluster 1											
Fruit	90.5 (73.2)	2.40	0.27	0.58	0.38	0.34	0.53	3.51	1.29	0.59	10.9
Grains and pasta	75.7 (42.5)	21.3	36.1	23.7	22.8	13.9	20.8	55.1	28.6	28.9	18.8
Legumes and pulses	78.6 (48.3)	35.3	30.0	40.5	31.3	37.9	21.5	70.5	33.2	48.1	11.1
Nuts and seeds	26.2 (7.92)	24.0	49.3	33.4	27.4	18.2	29.6	58.8	35.4	40.1	16.2
Potatoes, kumara and taro	32.0 (29.0)	2.75	6.66	4.25	2.82	3.86	3.00	6.07	3.62	4.09	9.13
Sugar and sweets	26.8 (7.98)	5.85	0.81	0.66	0.61	0.51	0.58	1.36	0.75	0.75	7.41
Vegetables	26.5 (31.7)	5.96	7.94	7.40	3.22	3.04	5.06	11.4	6.42	4.29	2.61
Yeast and condiments	22.1 (9.62)	13.0	26.3	24.1	14.6	19.5	11.8	24.3	14.3	17.7	3.75
Cluster 2											
Fruit	86.9 (74.8)	3.70	0.62	3.29	1.42	1.16	2.22	8.23	3.67	2.30	7.38
Grains and pasta	88.0 (41.9)	19.9	35.6	24.5	24.0	12.8	21.5	56.8	29.5	29.6	19.0
Legumes and pulses	73.7 (54.3)	29.7	23.9	33.1	25.3	30.9	17.9	58.9	27.6	39.2	9.38
Nuts and seeds	26.0 (8.23)	21.1	46.5	29.3	24.9	16.2	26.3	51.1	31.2	35.7	14.0
Potatoes, kumara and taro	51.3 (26.3)	3.53	8.83	5.76	3.77	5.00	3.92	8.08	4.77	5.40	10.8
Sugar and sweets	19.3 (10.9)	2.93	0.80	0.48	0.48	0.32	0.46	1.31	0.65	0.61	4.95
Vegetables	54.0 (26.9)	5.02	7.30	7.26	5.24	3.97	5.55	8.37	5.93	5.52	2.66
Yeast and other condiments	23.8 (10.4)	7.26	12.8	11.4	6.96	9.33	5.60	11.3	6.75	8.36	1.97
Cluster 3											
Fruit	0	0	0	0	0	0	0	0	0	0	0
Grains and pasta	70.5 (40.2)	12.8	21.7	15.7	14.7	8.76	13.9	31.7	17.7	17.7	16.2
Legumes and pulses	55.4 (40.9)	25.1	16.1	26.1	20.0	25.2	14.0	50.2	22.7	32.2	8.05
Nuts and seeds	26.2 (7.89)	17.7	33.1	22.4	20.6	13.0	18.3	39.1	22.6	29.7	13.9
Potatoes, kumara and taro	75.0 (0)	5.36	14.5	9.23	6.13	8.38	6.53	13.2	7.87	8.9	15.0
Sugar and sweets	25.0 (12.2)	3.68	1.37	0.81	0.82	0.54	0.77	2.23	1.11	1.04	5.91
Vegetables	3 (0)	3.77	7.44	6.73	3.08	2.25	4.77	6.70	4.97	2.95	0.71
Yeast and other condiments	0	0	0	0	0	0	0	0	0	0	0

1 Minimum daily requirement: protein was based on EAR (differentiated by sex and age), IAA was based on Table 1 and energy was based on minimum energy requirement with the consideration of PAL.

SD, standard deviation, Tryp, Tryptophan; Thr, Threonine; Leu, Leucine; Lys, Lysine; Met, Methionine, Cys, Cystine; SAA, sulphur amino acids; His, histidine.

### 3.2 Assessment of protein and IAA intake after optimisation

**Figure 2:**
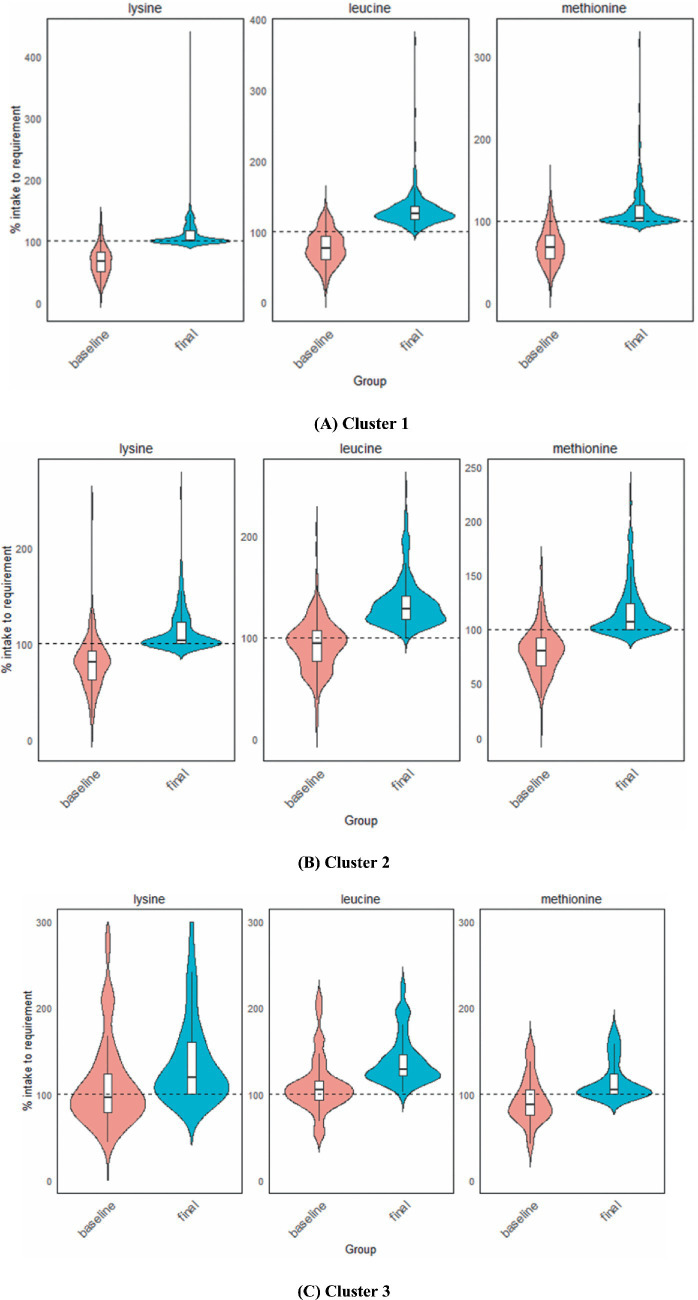
Comparison of adequacy in three selected IAAs, leucine, lysine, and methionine, at baseline (unmodified diets) and final (modified) intakes following diet optimisation in cluster 1 **(A)**, cluster 2 **(B)**, and cluster 3 **(C)**. The dotted line at 100% indicates the average minimum daily requirement for energy, protein, and each IAA. Each violin plot with its box plot represents the overall distribution of intake in each cluster, with wider portions of the violin plot representing a larger proportion of hypothesised intake to daily requirement. The top and bottom of the violin plots correspond to the maximum and minimum observed values of the cohort, smoothed by a density estimate. The horizontal line in the box plot represents the median (50th percentile) of the data with the interquartile range, defined by the 25th and 75th percentiles (lower and upper edges of the box plot, respectively). The lines extending from the percentiles represent the range (from the smallest to the largest non-outlier values), within 1.5 times the IQR. As the violin plots represent kernel density estimates, proportions of these plots may appear to fall below the 100% requirement. However, all successfully modified diets meet at least 100% of protein and all IAAs, without exceeding the upper boundary for energy (see Supplementary Figure S1).

Optimisation can resolve all shortfalls for protein and each IAA, achieving the daily requirements for these constrained nutrients through food additions from each individual’s food record, across all three clusters for most daily diets. In Figures 2A–C, comparisons of the baseline and final diets for three selected IAAs, lysine, leucine and methionine are shown for clusters 1–3. Results for energy, protein and other IAAs can be found in Supplementary Figure S1. The violin plots illustrate the cohort distribution of intake relative to daily requirements. Requirements are demarcated at 100% of the y-axis and plot points above the 100% line indicate fulfilment of the daily requirement for the IAA.

Cluster 1 had the largest proportion of baseline daily diets that fell below the requirement for each IAA compared with other clusters (Figure 2). This is most apparent for leucine (83.6%), lysine (92.7%) and methionine (90.9%). For the baseline diets in cluster 2, similarly large proportions of daily diets fell below requirements, especially for lysine and methionine, at 84.1 and 87.1%, respectively, but less so for leucine, where 62.6% were below requirements. In contrast, baseline diets in cluster 3 were closer to daily requirements for all nutrients, with fewer diets falling below the requirements, even for leucine (41.7%), lysine (52.8%) and methionine (69.4%).

Table 4 presents the mean protein and IAA quantity of all clusters relative to individual BW at baseline and in final diets. In the final diets, the EAR was achieved for all individuals and satisfied the model constraint for protein requirement. Most individuals were within the AMDR for protein of 10% to 35%, but 14.1% and 9.2% in clusters 1 and 2, respectively, were below this range, indicating a smaller contribution of protein to daily energy. The mean daily protein consumed in grams per individual increased from 39.8 g (11.8) at baseline to 60.9 g (16.0) in cluster 1. For cluster 2, the increment was from 51.4 g (15.3) to 68.6 g (16.7), and from 76.7 g (19.5) to 88.9 g (23.4) in cluster 3.

**Table 4:** Mean daily protein and IAA content (SD) in the baseline and optimised diets, relative to individual body weight (BW) across clusters 1 to 3.

Protein			IAAs (mg/kg BW)							
	Protein (g/kg BW)	AMDR in % of energy from protein	Cystine	Histidine	Leucine	Lysine	Methionine	SAA	Tryptophan	Threonine
Cluster 1										
Baseline	0.59 (0.17)	10.2 (2.43)	6.99 (2.29)	10.9 (3.41)	30.2 (9.30)	20.0 (6.99)	6.92 (2.28)	13.9 (4.39)	5.03 (1.63)	15.2 (4.75)
Final	0.90 (0.23)	12.6 (2.98)	11.6 (3.38)	18.4 (4.23)	50.6 (9.75)	33.8 (8.99)	11.5 (2.71)	23.1(5.73)	8.04 (1.84)	24.7 (4.8)
Cluster 2										
Baseline	0.72 (0.23)	11.7 (3.41)	8.32 (2.53)	13.0 (3.67)	36.1 (10.2)	23.8 (7.80)	8.04 (2.21)	16.4 (4.55)	5.68 (1.83)	17.5 (4.57)
Final	0.96 (0.23)	13.5 (3.45)	11.9 (2.79)	18.9 (3.5)	52.2 (9.54)	34.3 (7.03)	11.7 (2.52)	23.6 (4.94)	8.07 (2.17)	24.9 (3.8)
Cluster 3										
Baseline	1.02 (0.31)	17.6 (7.21)	11.1 (3.83)	16.8 (5.58)	43.3 (12.9)	33.4 (15.0)	9.30 (2.69)	20.3 (6.20)	5.06 (2.07)	21.1 (6.23)
Final	1.18 (0.34)	17.7 (4.93)	13.5 (3.76)	21.1 (5.17)	54.3 (11.2)	41.0 (14.5)	11.6 (2.28)	25.1(5.72)	6.51 (1.97)	26.2 (5.28)

AMDR, Acceptable Macronutrient Distribution Range, SD, standard deviation; BW, body weight; SAA, sulphur amino acids.

**Figure 3:**
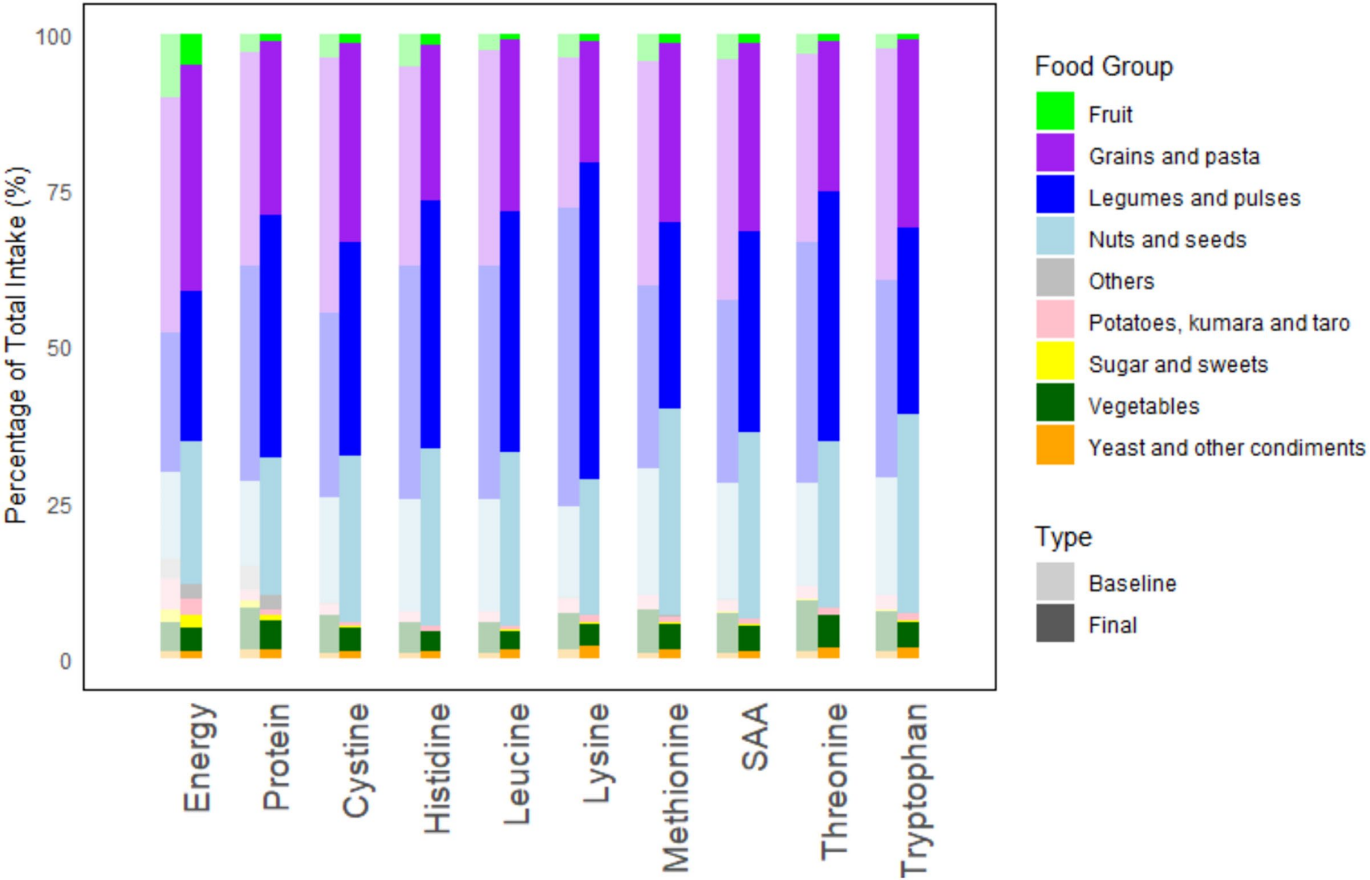
Comparison of the final diet with the baseline diet for cluster 1. Stacked bar graphs represent the contribution of each FG to energy, total protein, and IAAs as a percentage of the total intake. “Others” consist of alcoholic and non-alcoholic beverages. “Type” indicates that baseline diets are in bar graphs that are lighter in color.

A decrease in the contribution of energy, total protein, and all IAAs from fruit, vegetables and grains was observed when shifting from the baseline to the final diet, largely due to substantial increases in other FGs. Nuts, seeds and yeast products contributed more to energy, total protein and all IAAs from baseline to final diets. In contrast, apart from increases in the contributions of protein and lysine, the contributions by legumes and pulses remain relatively unchanged in baseline and final diets. Changes in FG intake were similar across clusters. Hence, the comparison of the final with the baseline diet is shown only for cluster 1 (Figure 3). Supplementary File S1 details changes in food groups across all clusters.

Grains and legumes together account for the largest proportion of total food mass in roughly a 1:1 ratio. When comparing how the total weight of each FG changed from the baseline to the final diet, FGs that had at least a 10% increase in all clusters were “legumes and pulses” and “nuts and seeds.” FGs that had a reduction in total weight (noting that this only occurred in cases where foods were required to be removed from the diet to provide residual energy capacity) were mainly energy-dense, from “potatoes, kumara and taro,” and “others,” which consisted of alcoholic and non-alcoholic beverages such as fruit juices and sugar-sweetened beverages (SSBs).

### 3.3 Sensitivity analyses

When the upper boundary for energy was reduced from + 20% to + 10% and + 5% of the EER_min_, the number of unsolved optimisation cases increased (*n* = 60 and *n* = 77, respectively) as cases which exceeded the + 20% threshold inevitably also exceeded tighter energy boundaries. However, the mean weight of additional foods required to achieve adequacy among the solvable cases remained similar, indicating those solutions which were feasible were largely unaffected. Importantly, 91.1% of solvable days required substantially less than the + 20% energy margin. The energy boundary of + 20% should be interpreted as a flexible safety margin to improve model feasibility but not a prescriptive increase of energy intake in practice.

Sensitivity analyses were conducted on the 52 daily diets for which no feasible optimisation solutions were identified. This involved progressively increasing the energy allowance (EER_max_) and removing serving size restrictions. Even when the allowable energy intake was increased to twice that of the EER_min_—a level considered unrealistic in habitual diets—feasible solutions remained unattainable for most cases. This finding indicates that the food choices in these diets are insufficient to ensure adequate protein quality and likely require substantial restructuring of dietary patterns. Only five individual diets achieved at least 70% coverage of the limiting nutrient shortfall. This was only possible because these diets had fewer nutrient gaps and smaller shortfall magnitudes, making it more feasible to approach adequacy under relaxed energy constraints and within the limited food options available in the baseline diets.

Predictably, removing serving-size restrictions (while retaining energy constraints) resulted in the prioritisation of foods in the habitual diet with the highest nutrient density. Legumes, pulses, nuts and seeds were preferentially selected to address shortfalls, and once these were maximised, lower protein-dense foods that were also low in energy contribution (e.g., green vegetables) were incorporated in large quantities. An alternative approach was explored for the 199 problematic cases in which the model could select foods from any diet in the cohort and add them to the original diets. Extruded plant proteins—notably pea, faba bean and hemp isolates found in protein powders—as well as spirulina and various nuts and seeds (hemp, pine nut, walnut, sesame) were frequently selected in this approach. These foods were also commonly found in the daily diets of those which had no protein or IAA shortfalls (*n* = 264). Nutrient shortfalls were resolved with small increases in mean energy contribution of 0.71 MJ (SD = 0.81) using this approach, highlighting the value of these protein-dense PB foods in addressing protein quality shortfalls while modestly increasing overall daily energy contribution. Similarly, testing the removal of protein dense PB isolates resulted in 11 more daily diets that had no feasible solutions, with 8 cases from daily diets that were meeting or above the EER_min_. Taken together, there is a potential that baseline diets can be largely maintained, provided that these protein-dense foods are included into the diets as supportive additions to improve protein quality, and modest changes in overall energy intake are acceptable.

### 3.4 Assessment of micronutrient intake after optimisation

The influence of food addition on the quantity of vitamin B12, calcium, iron, iodine, zinc, ALA, sodium and dietary fibre was examined post-optimisation. More than 90% of daily diets met or exceeded the daily requirements for dietary fibre, iron and zinc in all clusters. This should be interpreted with caution, as adequacy was assessed against the EAR only, without broader adjustments for bioavailability, which is relevant for iron and zinc, especially in a PB diet. In addition, the reliance on the EAR cut-point approach may overestimate adequacy for nutrients with skewed requirement distributions, such as iron among menstruating women, for whom a probability analysis would be more appropriate. Many optimised diets remained below the minimum intakes for calcium, vitamin B12 and iodine across all clusters, particularly in cluster 1. Hence, food additions based only on constraints in protein, protein quality and energy content cannot correct these micronutrient shortfalls.

The mean intake and percentage of daily diets meeting the requirement for each micronutrient, however, increased from baseline to final diets, indicating that dietary optimisation not only reduced shortfalls in protein quality, but also provided a small benefit for some micronutrients of concern. Interestingly, the dietary clusters stratified by protein intake at the EO level also reflected similar trends in micronutrient intake, with cluster 1 exhibiting the lowest nutrient intake, relative to the requirements. Table 5 illustrates the mean content of these nutrients and the percentage of the daily diets that meets the requirement in the optimised diets.

**Table 5:** Comparison of the mean intake of micronutrients (SD) and percentage adequacy^1^ between baseline and final diets.

	ALA (g)	Dietary fibre (g)	Calcium (mg)	Iron (mg)	Iodine (μg)	Sodium (g)^2^	Vitamin B12 (μg)	Zinc (mg)
Cluster 1								
Baseline								
Mean intake	1.48 (1.3)	35.7 (12.7)	673.4 (367.5)	14.1 (5.7)	101.5 (95.5)	2.08 (1.0)	1.44 (2.6)	7.71 (4.7)
Adequacy^1^ (%)	55.3	77.6	20.5	92.7	35.6	46.6	20.5	49.8
Final								
Mean intake	3.62 (3.78)	43.8 (13.2)	799.4 (376.3)	19.1 (6.35)	117.7 (103.9)	2.45 (1.16)	1.87 (3.34)	11.5 (6.92)
Adequacy^1^ (%)	75.3	94.1	33.8	98.6	46.6	61.6	26.0	94.1
Cluster 2								
Baseline								
Mean intake	1.92 (1.8)	39.6 (12.5)	769.0 (374.4)	16.2 (5.91)	144.7 (479.8)	2.28 (1.5)	1.41 (1.8)	8.89 (2.5)
Adequacy^1^ (%)	63.1	87.2	31.8	95.4	35.9	47.7	22.6	60
Final								
Mean intake	3.34 (3.59)	45.1 (13.7)	869.1 (349.1)	20.2 (6.96)	154.2 (479.1)	2.55 (1.47)	1.62 (1.78)	11.9 (3.16)
Adequacy^1^ (%)	75.9	94.4	45.1	99.5	43.1	59.0	26.7	91.8
Cluster 3								
Baseline								
Mean intake	1.59 (1.8)	35.8 (12.8)	838.4 (445.9)	18.9 (7.23	134.9 (105.1)	2.50(1.4)	4.72 (11.1)	10.5 (3.3)
Adequacy^1^ (%)	47.2	77.8	33.3	94.4	55.6	52.8	44.4	72.2
Final								
Mean intake	2.43 (2.18)	40.2 (13.8)	903.4 (417.6)	21.7 (8.07)	133.5 (103.3)	2.46 (1.16)	5.32 (13.4)	12.2 (3.57)
Adequacy^1^ (%)	61.1	88.9	41.7	100	58.3	55.5	47.2	77.8

ALA, alpha-linolenic acid; SD, standard deviation.

1 Percentage adequacy describes the percentage of daily diets that meet or are above the daily nutrient requirement, at the EAR level, listed in Supplementary Table S2.

2 For sodium, the percentage indicates the proportion that has exceeded the recommended sodium intake of 2 g per day.

The mean contribution of nutrients as a percentage of the daily requirement by each added FG is presented in Supplementary Table S3. This analysis was used to identify the key food sources responsible for high contribution of specific micronutrients. For example, relatively large quantities of spirulina in large quantities (15 g) in the baseline diet, fortified yeast products, and fortified PBMA were the primary contributors to vitamin B12 intake. ALA contribution was mostly from nuts and seeds, and grain foods. Sodium contributions were mainly from sauces and condiments, protein powders and PBMAs. At least 50% of cases in the final diets across all clusters exceeded the recommended intake of 2000 mg although sodium intake in baseline diets were already high.

Despite the mean calcium content in modified diets approaching the EAR of 840 mg across all clusters, there was substantial variation. One case from cluster 1 was above the UL for calcium, which was already above the UL at baseline. Similarly, large variations were observed for iodine, with two cases from cluster 2 exceeding the UL, but this was also observed in baseline diets of these individuals due to seaweed consumption. For zinc, one case in cluster 1 was above the UL, and was already the case at baseline due to the consumption of protein powder. Three cases from cluster 2 were above the UL for iron in the final diets and were close to, but not above, the UL at baseline. The addition of iron-rich nuts, seeds and legumes during optimisation increased the dietary iron and ALA content.

Although micronutrient adequacy was not prioritised as a primary optimisation constraint in this study, additional analyses were conducted to characterise which micronutrients were most difficult to achieve from habitual vegan diets. Specifically, for each daily diet in which these micronutrients were present in foods already consumed, and where additional energy intake was theoretically feasible, the maximum achievable adequacy was quantified under energy and serving-size constraints. This analysis was intended to estimate the highest level of micronutrient adequacy achievable from foods alone. The mean achievable adequacy was lowest for vitamin B12, calcium and iodine, reaching only 34.3%, 38.9% and 42.2% of requirements, respectively.

When micronutrients were incorporated as optimisation constraints along with protein and IAAs, the number of feasible solutions declined substantially. Only 131 of 502 daily diets were successfully resolved, compared with 303 diets achieved in the previous optimisation. Moreover, the optimised solutions required approximately twice as many added food items and total added food weight, indicating substantial dietary change. This degree of modification could also compromise the acceptability of the optimised diets and reduce their real-world feasibility. As mentioned, specific foods such as spirulina, yeast products and novel PBAs emerged as key contributors to these limiting micronutrients, the latter two could be related to fortification strategies in these foods.

## 4 Discussion

The main objective in this study was to address inadequate intakes in protein and IAA found in the current diets of a vegan cohort in NZ. Mathematical optimisation resolved the nutritional shortfalls of 90% of the daily diets while minimising the weight of food added, with the use of food items found in each individual’s existing diet. Consequently, the model generated outputs of the weight (g) and nutrient contribution for total protein, energy, and IAAs. At the same time, some beneficial impact to certain micronutrients was observed after diet optimisation.

### 4.1 Diet optimisation and key findings

The weight of food added must be guided by both the energy and serving-size constraints as these parameters ensure that optimised diets are feasible in reality. Designing optimal vegan diets can be challenged by the need to correct nutrient shortfalls while simultaneously maintaining energy intake within limits—two constraints that may be incompatible within the baseline diet (3, 37). This was the case in this study for individuals who were already meeting or above the maximum boundary of EER but had shortfalls for protein and/or AAs.

Substitution of foods in step 6 of Figure 1 enabled 147 daily diets, out of an unsolved 199, to meet protein and IAA shortfalls, while keeping below the EER_max_. The majority of removed foods were fruit, beverages (alcoholic, SSBs and fruit juices), grains and potatoes. Eliminating these foods provided residual energy capacity to accommodate the addition of more protein-dense foods, from legumes, pulses, nuts and seeds—FGs which had a larger increase in total weight in the optimised diets as compared to other food groups. This observation was aligned with a Swedish diet optimisation study, where pulses, meat and dairy substitutes (pea, soy and mycoprotein-based products), but not nuts, had a ten-fold increase compared to the baseline diet (16). As observed in other diet optimisation studies (17, 38), legumes, nuts and seeds are noted to be good sources of lysine and likely contributed to narrowing the lysine shortfall in this vegan cohort (Figure 3).

Nuts and seeds were also key FGs added in the optimised diets of this study, contributing to total protein and other IAAs (leucine and methionine) in higher quantities per gram compared to legumes and pulses, albeit with higher energy contribution. Nuts as valuable protein sources were highlighted in food replacement scenarios among pregnant women in Australia and identified to contribute at least twice the protein of tofu, legumes and pulses (39). Compared with the current Danish diet, the *NutriHealthGHGE* diet—an optimised diet modelled to meet nutritional adequacy, health and greenhouse gas emissions (GHGE) goals—showed substantial shifts in protein source composition. To compensate for the displacement of ruminant meat, the intake of nuts and seeds showed a marked increment from 6 to 20 g per day (+230%), alongside increases in other animal-sourced proteins (fish, eggs, and poultry) (40).

Identifying energy-dense, low-protein foods for removal was a pragmatic strategy to explore the trade-off between protein quality and energy constraints. This approach highlighted a critical challenge in PB diet optimisation. When the baseline diets contain foods that are relatively energy-dense but protein-poor (as exemplified by the problematic cases in this study), substantial dietary change may be required to accommodate food substitutions with higher protein density. Our approach was computationally simple but could also be reductive. While many removed foods were discretionary, some contributed micronutrients of importance, such as fruits and PB beverages, for which alternative sources may be limited.

Given that fruits and vegetables are an excellent source of vitamin C (41) and other essential micronutrients, these foods are central components of FBDGs around the world (40, 42, 43). Hence, the reduction of 20%–30% weight of fruit in the final diets of the clusters in this study is a concern. As an indication, vitamin C intake reduced from baseline to optimised diets, although the final intake was still above the Recommended Dietary Intake (RDI) (26) (Supplementary Table S4). Vegetables were less affected, with a decrease of less than 7% in weight across the final diets of the clusters. The broader limitation of the substitution approach was the complete removal of certain foods, applied only in the problematic cases. While this was consistent with the study’s aim of examining how vegan diets may be optimised within energy boundaries, a more sophisticated approach could incorporate partial displacement of foods and/or incorporate additional constraints to better reflect realistic dietary modification. This is further explained in the strengths and limitations section.

Given the high protein density and protein quality of legumes, nuts and seeds, and their complementary effect with grains to achieve protein adequacy, the proportion and balance of these foods within the contexts of vegan diets warrant further investigation. The best ratio of grain to legume in plant-based protein complementation remains unclear and may vary across different foods. For example, substituting 200 g of cooked chickpeas with 120 g of cooked lentils improves the protein quality score due to the higher protein content of lentils (44). This demonstrates variation in protein quality even within one FG. An approximate 1:1 ratio was observed to be sufficient in the optimised diets of this study, but a 2:1 ratio may be more effective for optimal protein quality (15). More experimental verification or modelling at the meal level is therefore necessary to establish more precise ratios of these foods. Food complementation at the meal level is currently explored in our subsequent research via The Vegan Protein Quality (VPQ) tool, a web application that allows users to choose mixtures of PB meals to meet individual energy, protein and IAA requirements (45).

The post-optimisation analysis of dietary fibre, ALA, calcium, zinc, iron, iodine, sodium and vitamin B12 were examined. The most concerning nutrients at risk of deficiencies were vitamin B12, iodine and calcium, where the percentage of daily diets achieving adequacy was approximately 50% or below. Results were generally poorer in clusters 1 and 2. PB foods are not always reliable sources of bioavailable versions of these micronutrients. Limited amounts of vitamin B12 for example is obtained from seaweed, protein powder and yeast products (46)—foods found in the baseline diets of this vegan cohort. Spirulina is a rich source of vitamin B12 (30) and contains the largest quantity of the micronutrient at 3 mg per gram in the vegan dietary records of this study. It is also a high contributor of iodine (22 mg per gram). Concomitant improvements in micronutrient intake were observed with the addition of protein-dense foods. For example, the addition of tofu, soy-based foods and seeds simultaneously increased calcium intake in the optimised diets.

When the optimisation was constrained to meet the EAR for selected micronutrients, fortified foods in the forms of PBMAs, PB beverages and protein powders were the largest contributors to several micronutrients, particularly vitamin B12, iodine, zinc and iron. Similar observations were reported in other studies. For example, the inclusion of a fortified soy drink in a vegan diet scenario in the Netherlands increased calcium intake to meet requirements (42). Similarly, optimised vegan diets in Sweden achieved calcium and vitamin B12 adequacy through high quantities of fortified PB alternatives (16). However, these benefits must also be considered alongside potential trade-offs. Depending on formulation, these foods may have high caloric density and contribute to excess sodium, as compared to traditional PB sources (47–49). We highlight the important role of food formulation and product innovation in developing fortified PB foods that can support the provision of bioavailable micronutrients and utilisable protein.

A substantial reduction in the number of feasible solutions after including micronutrient constraints was an expected observation. Few foods within the existing baseline diet could meet protein quality and all micronutrient requirements within the required energy boundaries. Given the challenges of attaining nutrient adequacy in this vegan cohort for vitamin B12, iodine and calcium from whole foods alone, supplementation is likely needed to fill remaining gaps. Without appropriate documentation of supplement use in the baseline diets and its inclusion in diet optimisation, total micronutrient intake in the cohort may be underestimated. In support, Storz et al. (50) observed adequate vitamin B12 status among vegans due largely to >90% of the cohort consuming a daily median intake of 250 mg of a vitamin B12 supplement. Supplementation expenditure is sizeable in vegan populations, and about 2 to 4 times higher than in omnivores and lacto-ovo-vegetarians, as indicated by one study on German vegans (51). Despite its importance, vegan dietary studies (including this present study) often exclude supplement use in the food recall data (52, 53). Reliable records of supplement usage in vegan dietary records could improve modelling outcomes for micronutrient adequacy in vegan diets.

Across all clusters, a high intake of dietary fibre was also introduced in the optimised diets, with more than 90% of individuals in all clusters consuming quantities above the requirements. While dietary fibre intake is associated with several health benefits, including enhanced satiety, which has a beneficial role in appetite regulation, it can impair nutrient digestion and utilisation by the body (54). The introduction of large quantities of dietary fibre in the modelled diets may present a practical challenge in reality, as overall food intake may become limited due to satiety (39, 55). Hence, this may restrict the consumption of protein, IAAs and essential micronutrients.

### 4.2 Strengths and limitations

A key strength of this study is correcting protein quality shortfalls at the individual-level by incorporating calculations of energy, total protein and IAA requirements based on each participant’s BW, sex, age and PA. Another strength is the adjustment for protein and IAA digestibility of plant foods, with currently available TID coefficients, thus providing a more accurate estimation of the quantity of utilisable protein in the diet. Compared to population-level modelling, this individualised approach could potentially improve the acceptability and adherence to dietary interventions aimed at improving the protein quality of vegan diets (56).

The limitation of the model is its dependence on foods within the individual’s existing dietary records. For example, spirulina provides a supply of several micronutrients and protein, at a low energy density. However, it was not commonly consumed within this vegan cohort and additional food preparation may be necessary to improve its palatability. More broadly, the usefulness of this optimisation approach is tied to the diversity and nutritional quality of the baseline diet. For diets characterised by energy-dense and nutrient-poor options (in the case of the 199 problematic cases), modelling using benchmarking of peer diets may be more optimal to devise diets with a favourable balance of “more-is-better” versus “less-is-better” nutrients (4). By leveraging the dietary habits of peers within the same population, such approaches enable modifications of less optimal diets using culturally appropriate alternatives to improve both nutritional quality and feasibility (4, 9). However, within the context of vegan diets, this may be challenging in practice, due to the limited availability of diverse, nutritionally adequate vegan dietary records in the population to serve as peer benchmarks.

Diet variety was considered by introducing maximum serving limits of each food group, as guided by NZ dietary guidelines (29) and disallowing duplicated selections for each food item. A similar approach that applied serving-size constraints, as guided by the Australian Dietary Guidelines (26), was adopted in an LP optimisation exercise for various diet scenarios in Australia (6). By placing limits on the daily intake of each food item, the model ensured that some diversity was introduced to the diet while keeping to a practical volume of food item. However, one limitation of this approach is that more granular serving-size constraints are required to reflect differences across food types. For example, the model permitted servings of up to 150 g for legumes and pulses (Table 1), but is less applicable for legume-based protein isolates, which are typically consumed in quantities of 20 to 35 g per serve (57). This highlights the need for more food-specific serving-size guidelines within our future models to better capture realistic consumption patterns and improve the validity of dietary recommendations.

One limitation observed in the current model is that during substitutions, entire baseline food items are removed rather than having their portion sizes reduced or displaced by alternative foods. This means that all nutrients contributed by that food are also eliminated. Although the model may introduce the same food at a smaller serving-size, perhaps a more effective approach might involve modifying the food’s recipe or formulation to improve the protein quality, rather than removing it completely. Among the 199 problematic cases, 41 cases which had feasible solutions ended up with diets that were below the EER_min_ (Supplementary Figure S1, energy panel; whisker of box plot extending below line of requirement**)**. This suggests that removing the top 25% and 50% of energy-dense foods may be too restrictive in these scenarios, so enabling the partial removal rather than full elimination of some foods could be a more intuitive and realistic approach, a learning point used to inform the meal optimisation model (the VPQ tool). While the 25% and 50% thresholds used were arbitrary, further empirical testing is needed to determine optimal cut-off points for dietary feasibility. A displacement model could simultaneously reduce portions of energy-dense foods while adding alternative foods, thus being closer to real-world food substitution. Such a framework would be more complex and require assumptions for how foods can be partially displaced (e.g., 17 g of white rice may not be realistic in practice) and how much dietary change is acceptable. Aligned with the overall aim of investigating tensions between energy intake and protein density, the present approach, although simpler, is more transparent in identifying foods that are less efficient providers of utilisable protein.

Notably, the EAR for protein was applied instead of the RDI for protein, which may underestimate the total protein intake needed to meet adequacy in our optimised solutions. However, post-optimisation evaluation indicated that among individuals whose protein intake remained below the RDI, the majority still met the AMDR for protein (10% to 35% of total energy intake), indicating that protein intake is proportional to total energy intake. In several cases, individuals had approached the upper limit of energy intake, indicating limited scope to further increase food intake without exceeding realistic energy constraints. Only a small proportion of cases (<5%) had sufficient capacity to increase food intake further to meet both the RDI and AMDR simultaneously. Hence, if the RDI was applied as the lower-bound constraint across the entire cohort would have resulted in the prescription of unnecessarily large food quantities, despite IAA intake no longer being limiting.

The feasibility of the solutions from this study follows the assumption that the added foods will be acceptable because they are obtained from the current diets of each individual (58). Additionally, the solutions are only reliable when dietary intake data is reported accurately. However, misreporting is a common limitation with the use of dietary records. There is also no measurement to determine satiety from the consumption of protein and fibre-dense foods (55, 59, 60), or validate the extent of acceptability by the cohort. Furthermore, the calculation of the optimal nutrient intakes by LP is dependent on the quality of the food and nutrient composition databases used in the optimisation (6, 37). Food items from the four-day food diaries may not be an exact match to the USDA food items used, and even with normalisation, will result in AA compositions that are not precise. In the absence of NZ AA composition data, this however appears to be the best substitute.

Defining “currently consumed foods” from the four-day records may underestimate the variety of foods consumed by individuals over longer periods. Yet, foods outside of the records also do not guarantee acceptability. The vegan diet is inherently restrictive in high-quality protein sources and the dataset captured most major FGs that feature in modern-day vegan diets. An alternative approach was explored where the model could draw from all available food sources. However, the optimisation consistently defaulted to a narrow set of food items for inclusion into everyone’s existing diets, such as isolates to maximise protein quality with small increases to energy, which offers an oversimplistic solution with a lack of diversity.

This study has optimised protein intake and protein quality with respect to daily requirements but has not considered how foods can be spread across the day. Distributing protein-dense foods in appropriate quantities across multiple EOs is better tolerated than consuming large quantities of these foods in one meal. This is a relevant area of study given the time-sensitive nature of AA metabolism, where the coordinated intake of diverse protein sources within defined temporal windows is crucial to allow synchronised availability of all AAs, for maximum metabolic utilisation (61, 62). To do so, appropriate meal reference guidelines for protein and IAA requirements are necessary, but this information is currently lacking in the literature. Nevertheless, it is important to establish the proportion of complementary plant foods that should be included per meal and calculate the quantities of IAAs that together, meet daily intake requirements and are continuously examined in our future research.

## 5 Conclusion

This study demonstrated the feasibility of improving utilsable protein intake of vegan diets with mathematical optimisation. Constraining the model to select foods that already exist within each individual’s diet goes some way to ensuring acceptability. Nuts, seeds, legumes and pulses emerged as superior sources of total protein, leucine, methionine and lysine—the most commonly limiting IAAs in the vegan diet of this cohort. When large deficits were present, the model had limited ability to resolve them. This is a key challenge in designing protein-adequate vegan dietary patterns. The presence or absence of high-quality protein (such as extruded/isolated plant proteins with high digestibility) can influence the success of dietary optimisation.

Eliminating all animal-sourced foods requires careful selection of diverse plant sources to meet protein adequacy. The consequence of prolonged deficiencies in the essential AAs can manifest in various metabolic disorders. While this study has shown how to improve protein adequacy for most individuals in the cohort, the shortfalls for calcium, vitamin B12 and iodine were not resolved from food additions showing that apart from protein quality, several nutrients are problematic in vegan diets and should not be neglected in diet planning. Food fortification and supplementation have key roles in reducing the risks of micronutrient deficiencies and are likely more relevant for certain populations such as theelderly and women of reproductive age, who have increased nutrient requirements.

## Data Availability

The original contributions presented in the study are included in the article/Supplementary material, further inquiries can be directed to the corresponding author.

## Glossary

AA: Amino acid
AI: Adequate intake
ALA: Alpha-linolenic acid
AMDR: Acceptable Macronutrient Distribution Range
BW: Body weight
Cys: Cystine
EAR: Estimated average requirement
EER: Estimated Energy Requirement
FAO: Food and Agricultural Organisation
FBDG: Food based dietary guidelines
FG: Food Group
GHGE: Greenhouse gas emissions
His: Histidine
IAA: Indispensable amino acid
IPAQ: International Physical Activity Questionnaire
IQR: Interquartile range
Leu: Leucine
LP: Linear programming
Lys: Lysine
Met: Methionine
MET: Metabolic equivalent task
MJ: Megajoules
NRV: Nutrient reference values
NZ: New Zealand
PAL: Physical activity level
PB: Plant-based
PBA: Plant-based alternative
PBMA: Plant-based meat alternative
SAA: Sulphur amino acids
SD: Standard deviation
TID: True ileal digestibility
Thr: Threonine
Tryp: Tryptophan
UL: Upper level
USDA: United States Department of Agriculture
