# Supplementary material for: Improving the protein quality of New Zealand vegan diets: an optimisation modelling approach incorporating energy constraints and diet acceptability
Source: Front Nutr. 2026 Apr 22;13:1807755. doi: 10.3389/fnut.2026.1807755 (PMC13143576; doi:10.3389/fnut.2026.1807755)
Supplement: Supplementary file 1 [file Table_1.docx]

# Supplementary Information

**Supplementary Table 1.**  Daily IAA requirement values in mg per kg of body weight per day (1)

| AA | His | Leu | Lys | Met | Cys | SAA^1^ | Thr | Tryp |
| --- | --- | --- | --- | --- | --- | --- | --- | --- |
| Reference  (mg/kg/day) | 10 | 39 | 30 | 10 | 4 | 15 | 15 | 4 |

Abbreviations: AA, amino acid; His, histidine; Leu, leucine; Lys, lysine; Met, methionine; Cys: cystine; SAA, sulphur-amino acids; Thr, threonine; Tryp, tryptophan

^1^ SAA comprises the sum of methionine and cystine

**Calculation of the Physical Activity Level (PAL)**

The International Physical Activity Questionnaire (IPAQ) was used to compute the daily PA. Briefly, IPAQ records the number of metabolic equivalent task (MET) minutes, which represents a multiple of the estimated resting energy expenditure. For example, 1 MET indicates energy expended at rest. 3.3 METS, 4 METS and 8 METS represent energy expended during walking, moderate physical activity (PA) and vigorous PA respectively (2). The PAL was then calculated by adding 1 MET to the sum of the average MET values for each PA, weighted by the average number of minutes per week spent on that activity (3, 4), as shown:

[1]$PAL=1+\frac{\left[ \mathrm{Walk}\left( \mathrm{mins} \right) \times3.3+Moderate Activity \left( \mathrm{mins} \right) \times4 +Vigorous Activity \left( \mathrm{mins} \right) \times8 \right]}{10080 mins (per week)}$

**Supplementary Table 2**. Minimum daily requirements and upper limits of nutrients (5)

| Nutrient | Sex | Age | AI / EAR per day | UL / day |
| --- | --- | --- | --- | --- |
| ALA | Male | 19 and above | 1.3 g | - |
|  | Female | 19 and above | 0.8 | - |
| Dietary fibre | Male | 19 and above  19 and above | 30 g | - |
|  | Female |  | 25g | - |
| Vitamin B12 | Male and female | 19 and above | 2.0 µg | - |
| Calcium | Male | 19 to 70 | 840 mg | 2500 mg |
|  |  | Above 70 | 1100 mg |  |
|  | Female | 19 to 50 | 840 mg |  |
|  |  | Above 51 | 1100 mg |  |
| Iodine | Male and female | 19 and above | 100 µg | 1100 µg |
| Iron | Male | 19 and above | 6 mg | 45 mg |
|  | Female | 19 to 50  Above 51 | 8 mg |  |
|  |  |  | 5 mg |  |
| Sodium | Male and female | 18 and above | 2000 mg | - |
| Zinc | Male | 19 and above | 12 mg | 40 mg |
|  | Female |  | 6.5 mg |  |

Abbreviations: AI, adequate intake; ALA, alpha-linolenic acid; EAR, estimated average requirement; UL, tolerable upper intake level

- indicates no UL can be set for that nutrient


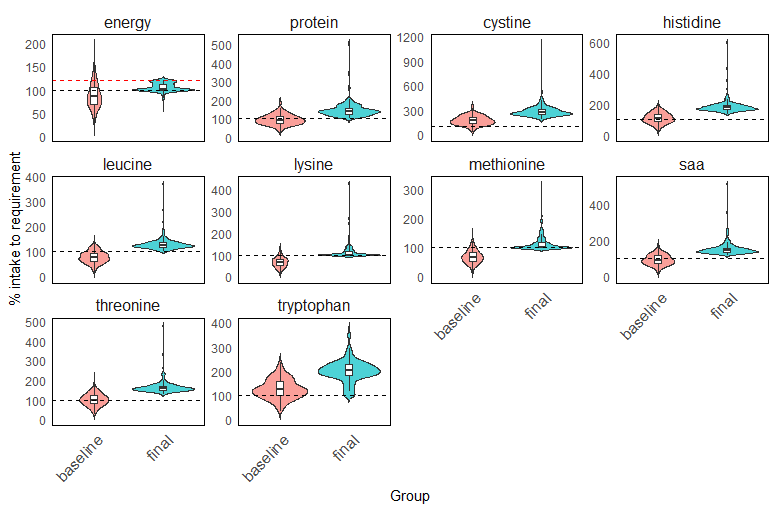


1. **Cluster 1**


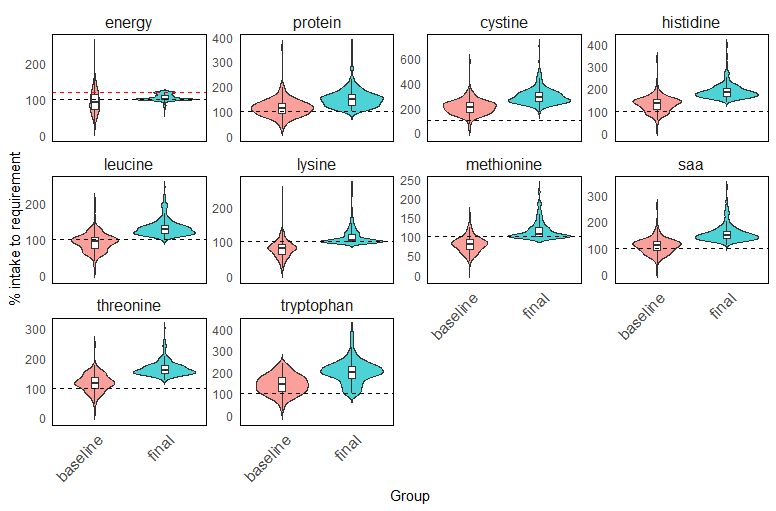


1. **Cluster 2**


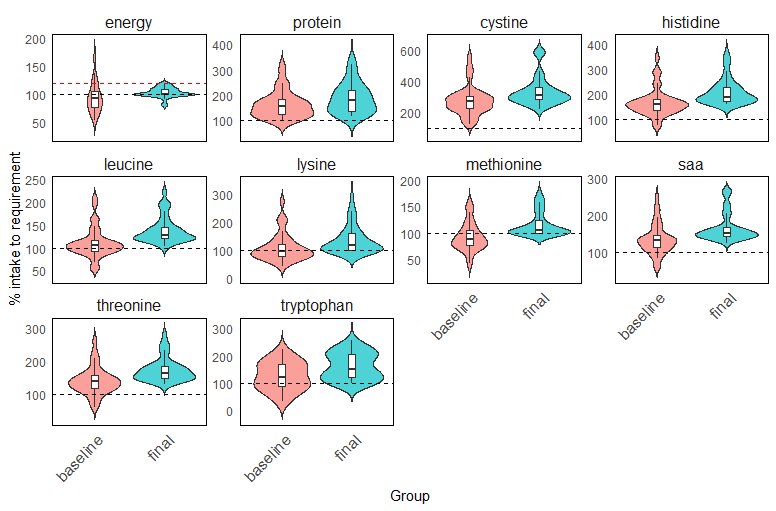


1. **Cluster 3**

**Supplementary Figure 1.** Comparison of adequacy in energy, total protein and each IAA at baseline (unmodified diets) and final modified intakes following diet optimisation in cluster 1 (A), cluster 2 (B) and cluster 3(C). The dotted line at 100 % indicates the average minimum daily requirement for energy and each nutrient. The dotted red line at 120% in the energy plot indicates the average maximum daily requirement for energy. Each violin plot with its box plot represents the overall distribution of energy or nutrient intake in each cluster, with wider portions in the violin plot representing larger proportion of daily diets. The top and bottom of the violin plots correspond to the maximum and minimum observed values of the cohort, smoothed by density estimate. The horizontal line in the box plot represents the median (50^th^ percentile) of the data with the interquartile ranges at 25^th^ and 75^th^ percentiles (lower and upper edges of the box plot respectively). The lines extending from the percentiles represents the range (smallest to the largest non-outlier values) which is within 1.5 times of the IQR.

**Supplementary Table 3.** Mean contribution of dietary fibre, ALA and micronutrients (%) for each added food group added after optimisation

| Cluster 1 | | | | | | | | |
| --- | --- | --- | --- | --- | --- | --- | --- | --- |
| Nutrient contribution to daily requirement ^1^ (%) | | | | | | | | |
| Food group | ALA | Dietary fibre | Calcium | Iron | Iodine | Sodium | Vitamin B12 | Zinc |
| Fruit | 0 | 19.3 | 8.46 | 33.6 | 0.29 | 1.85 | 0 | 4.76 |
| Grains and pasta | 30.5 | 18.7 | 6.73 | 29.3 | 18.3 | 13.6 | 2.84 | 19.2 |
| Legumes and pulses | 41.2 | 14.3 | 10.8 | 38.2 | 7.96 | 16.2 | 14.9 | 23.7 |
| Nuts and seeds | 144.0 | 12.3 | 4.14 | 21.2 | 0.98 | 1.28 | 0.91 | 15.1 |
| Potatoes, kumara and taro | 3.69 | 4.16 | 0.75 | 5.43 | 1.68 | 13.0 | 0 | 3.72 |
| Sugar and sweets | 1.99 | 4.52 | 1.60 | 19.4 | 1.24 | 0.35 | 1.54 | 7.44 |
| Vegetables | 5.27 | 9.31 | 2.59 | 18.6 | 28.1 | 1.41 | 196.3 | 27.6 |
| Yeast and condiments | 0.17 | 11.0 | 1.63 | 17.7 | 2.42 | 14.1 | 0 | 26.1 |
| Cluster 2 | | | | | | | | |
| Fruit | 7.33 | 18.2 | 7.69 | 25.2 | 0.04 | 3.85 | 0 | 2.32 |
| Grains and pasta | 15.7 | 15.8 | 5.99 | 28.0 | 12.5 | 13.1 | 1.10 | 14.3 |
| Legumes and pulses | 29.3 | 12.9 | 11.6 | 41.3 | 9.53 | 14.4 | 15.6 | 18.9 |
| Nuts and seeds | 102.1 | 10.3 | 3.73 | 21.6 | 1.22 | 1.40 | 0.76 | 14.8 |
| Potatoes, kumara and taro | 4.55 | 6.08 | 1.19 | 8.66 | 1.95 | 10.9 | 0 | 4.83 |
| Sugar and sweets | 1.56 | 4.08 | 1.29 | 21.6 | 1.03 | 0.19 | 1.43 | 5.50 |
| Vegetables | 13.5 | 5.61 | 3.03 | 9.00 | 4.68 | 3.16 | 26.2 | 4.17 |
| Yeast and condiments | 0 | 7.05 | 1.04 | 46.6 | 1.25 | 22.3 | 33.6 | 13.6 |
| Cluster 3 | | | | | | | | |
| Grains and pasta | 24.2 | 15.2 | 4.21 | 17.3 | 6.89 | 8.90 | 2.80 | 12.6 |
| Legumes and pulses | 22.8 | 9.11 | 10.2 | 34.3 | 7.97 | 12.4 | 9.20 | 12.0 |
| Nuts and seeds | 65.4 | 9.73 | 3.64 | 15.3 | 0.98 | 1.40 | 0 | 12.6 |
| Potatoes, kumara and taro | 5.77 | 8.50 | 1.92 | 13.8 | 3.90 | 26.4 | 0 | 5.31 |
| Sugar and sweets | 3.75 | 7.73 | 1.84 | 34.0 | 1.84 | 0.42 | 2.25 | 7.60 |
| Vegetables | 0 | 1.50 | 0.55 | 17.8 | 66.0 | 1.58 | 471.0 | 0.92 |

Nutrient contribution (%) is calculated as the total intake of each nutrient per food group as a percentage of the daily nutrient requirement (Table 4) at the EAR level.

**Supplementary Table 4**: Mean baseline and final intake, and percentage adequacy of other micronutrients across clusters 1 to 3.

|  | Vitamin A (mg) | Vitamin B6 (mg) | Niacin (mg) | Thiamine (mg) | Folate  (mg) | Vitamin C (mg) | Vitamin E (mg) | Potassium (mg) | Magnesium (mg) | Phosphorus (mg) | Selenium (mg) |
| --- | --- | --- | --- | --- | --- | --- | --- | --- | --- | --- | --- |
| Cluster 1 | | | | | | | | | | | |
| Baseline | | | | | | | | | | | |
| Mean intake | 864.32 | 2.09 | 13.4 | 1.47 | 363.4 | 120.2 | 15.6 | 3213.4 | 416.4 | 1083.9 | 39.9 |
| % adequacy | 34.2 | 72.6 | 54.8 | 70.8 | 53.9 | 81.7 | 85.8 | 53.9 | 82.2 | 96.3 | 13.7 |
| Final | | | | | | | | | | | |
| Mean intake | 834.7 | 2.20 | 18.1 | 1.90 | 454.5 | 107.6 | 24.5 | 3551.02 | 592.0 | 1525.6 | 54.2 |
| %adequacy | 32.0 | 83.6 | 84.5 | 89.5 | 72.1 | 78.5 | 94.5 | 71.2 | 98.2 | 100 | 37.9 |
| Cluster 2 | | | | | | | | | | | |
| Baseline | | | | | | | | | | | |
| Mean intake | 781.6 | 1.92 | 15.4 | 1.74 | 384.7 | 118.6 | 16.6 | 3252.8 | 479.7 | 1304.3 | 42.7 |
| % adequacy | 34.4 | 76.9 | 67.2 | 85.1 | 58.5 | 84.6 | 83.1 | 51.8 | 90.8 | 97.4 | 18.5 |
| Final | | | | | | | | | | | |
| Mean intake | 725.3 | 2.05 | 19.4 | 2.07 | 456.7 | 111.7 | 25.1 | 3450.4 | 627.4 | 1677.2 | 54.6 |
| % adequacy | 30.8 | 84.1 | 86.7 | 95.4 | 69.7 | 82.1 | 92.8 | 57.9 | 98.5 | 99.5 | 40.5 |
| Cluster 3 | | | | | | | | | | | |
| Baseline | | | | | | | | | | | |
| Mean intake | 723.1 | 1.86 | 17.4 | 1.60 | 430.4 | 77.6 | 19.4 | 2966.2 | 447.6 | 1394.4 | 53.2 |
| % adequacy | 27.8 | 75.0 | 83.3 | 77.8 | 66.7 | 63.9 | 91.7 | 41.7 | 83.3 | 100 | 22.2 |
| Final | | | | | | | | | | | |
| Mean intake | 727.5 | 1.87 | 21.1 | 1.83 | 459.2 | 76.5 | 26.8 | 3172.9 | 545.5 | 1631.9 | 59.2 |
| % adequacy | 27.8 | 75 | 88.9 | 88.9 | 69.4 | 66.7 | 88.9 | 50.0 | 91.7 | 100 | 47.2 |

References

1. FAO, WHO. Protein and amino acid requirements in human nutrition: report of a joint FAO/WHO/UNU expert consultation: World Health Organization; 2007.

2. Forde C. Exercise Prescription for the Prevention and Treatment of Disease: Scoring the International Physical Activity Questionnaire (IPAQ) Trinity College Dublin, The University of Dublin. [Available from: <https://ugc.futurelearn.com/uploads/files/bc/c5/bcc53b14-ec1e-4d90-88e3-1568682f32ae/IPAQ_PDF.pdf>.]

3. Piazza L, Ferreira E, Minsky R, Pires G, Silva R. Assesment of physical activity in amputees: A systematic review of the literature. Science & Sports. 2017;32(4):191-202.

4. FAO/WHO/UNU. Human energy requirements: report of a joint FAO/WHO/UNU Expert Consultation. Rome; 2004.

5. NHMRC. Nutrient Reference Values for Australia and New Zealand Including Recommended Dietary Intakes. Version 1.2. 2017.
